# Authentication of *Greek PDO Kalamata* Table Olives: A Novel Non-Target High Resolution Mass Spectrometric Approach

**DOI:** 10.3390/molecules25122919

**Published:** 2020-06-24

**Authors:** Natasa P. Kalogiouri, Reza Aalizadeh, Marilena E. Dasenaki, Nikolaos S. Thomaidis

**Affiliations:** Laboratory of Analytical Chemistry, Department of Chemistry, National and Kapodistrian University of Athens, Panepistimiopolis Zographou, 15771 Athens, Greece; kalogiourin@chem.uoa.gr (N.P.K.); raalizadeh@chem.uoa.gr (R.A.); mdasenaki@chem.uoa.gr (M.E.D.)

**Keywords:** Kalamata olives, *PDO*, non-target analysis, markers, QTOF-MS, PLS-DA

## Abstract

Food science continually requires the development of novel analytical methods to prevent fraudulent actions and guarantee food authenticity. Greek table olives, one of the most emblematic and valuable Greek national products, are often subjected to economically motivated fraud. In this work, a novel ultra-high-performance liquid chromatography–quadrupole time of flight tandem mass spectrometry (UHPLC-QTOF-MS) analytical method was developed to detect the mislabeling of *Greek PDO Kalamata table olives*, and thereby establish their authenticity. A non-targeted screening workflow was applied, coupled to advanced chemometric techniques such as Principal Component Analysis (PCA) and Partial Least Square Discriminant Analysis (PLS-DA) in order to fingerprint and accurately discriminate *PDO* Greek *Kalamata* olives from *Kalamata (or Kalamon) type* olives from Egypt and Chile. The method performance was evaluated using a target set of phenolic compounds and several validation parameters were calculated. Overall, 65 table olive samples from Greece, Egypt, and Chile were analyzed and processed for the model development and its accuracy was validated. The robustness of the chemometric model was tested using 11 Greek *Kalamon* olive samples that were produced during the following crop year, 2018, and they were successfully classified as Greek *Kalamon* olives from Kalamata. Twenty-six characteristic authenticity markers were indicated to be responsible for the discrimination of *Kalamon* olives of different geographical origins.

## 1. Introduction

Τhe olive tree (*Olea Europaea L.*) is cultivated mainly in the Mediterranean region and has diverged naturally to many cultivars [1]. Table olives, prepared from the olive fruit, are considered a highly functional food and their quality depends on the cultivar [2,3]. Eating table olives provides us with energy, vitamins, minerals, and a large variety of phenolic antioxidants, such as phenolic acids, phenolic alchohols, flavonoids, and secoiridoids, compounds known for their antioxidant, antiinflammatory, anticarcinogenic, antimicrobial, antidyslipidemic, antihypertensive, cardiotonic, laxative, and antiplatelet properties [3,4,5,6,7].

According to the International Agreement on Olive Oil and Table Olives, the table olive is defined as the healthy fruit of certain varieties of the cultivated olive tree, harvested at a stage of appropriate maturity and quality, to yield a well-preserved product after proper treatment [8]. Table olives are classified as green olives, turning color olives, and black olives depending on their ripening stage. Olive drupe processing is essential, aiming primarily at the degradation of the phenolic glycoside oleuropein, a compound that gives a bitter taste to the fruit, making immediate consumption impossible [7]. There are three types of commercial table olives: *Greek-style natural black olives*, *California-style black olives*, and *Spanish-style green olives*. The processing of the *Californian* and *Spanish* olives requires treatment with a diluted aqueous NaOH solution that changes the initial phenolic fingerprint of the drupe by hydrolyzing the bitter glucoside of oleuropein [9]. On the contrary, the production of the *Greek-style* table olives is more natural and does not involve the use of chemicals, apart from brine [9,10].

The Mediterranean countries constitute a highly developed olive market with many suppliers and buyers and a wide variety of table olive products. Despite the high competition, consumers are always looking for new and unique products that can satisfy their need for healthy, authentic natural products. The European Union has established regulations providing guidelines to maintain the Protected Geographical Indication (PGI) and the Protected Designation of Origin (PDO) to reassure that the products are closely linked to their botanical and geographical origins [11]. Olive products, under a PDO or PGI label, have a raised market value which makes them an attractive target for fraudsters [12,13].

As far as the Greek table olives are concerned, the most common olives belong to the *Kalamon*, *Chalkidiki*, *Throumbolia*, and *Konservolia* varieties. Among them, the *Kalamon* table olive is perhaps the most famous Greek variety, certified by the European Union designation of origin (*PDO Kalamata*), initially cultivated and adapted to the microclimate and the soils of Messinia from where it was spread to Laconia. It is a medium-grain variety, almond-shaped, with a pointed nose and a glossy and durable wristband. The skin of the ripe fruit acquires a deep black color, matures in November, and is prolonged in the case of overproduction. The PDO *Kalamata Olive* has been registered in the EU since 1996 [14] and is allowed to be used only for olives of the *Kalamon* variety produced in Messinia at Southern Peloponnesus, Greece. However, the term *Kalamata olives* has been widely misused by many countries outside the EU such as Egypt, Turkey, and even South American countries like Chile, instead of the correct term *Kalamata type olives* or *Kalamon olives*. In this respect, there is an emerging need for the development of authenticity tools that can guarantee the Greek *PDO Kalamata table olives* label, taking into account their high nutritional value and health properties as well as their economic importance for the Greek market.

Performing a thorough search of the literature makes it evident that there have been extensive studies concerning the authentication of Italian [15], Tunisian [16], Turkish [17], Portuguese [10], and Spanish [18,19] PDO and PGI table olives. The current state-of-the-art technology for the determination of bioactive constituents in foods has also been reviewed recently [20]. For the determination of the characteristic biomarkers in table olives, different methodologies have been applied, such as polymerase chain reaction (PCR)-based techniques [21], Nuclear Magnetic Resonance spectroscopy (NMR) [15], High Pressure Liquid Chromatography (HPLC) [17,22], or Gas Chromatography (GC) [18] coupled to either Mass Spectrometry (MS) [22] or UV detection [16,23].

However, studies concerning Greek table olives are very scarce. Published works mostly present the profiling of *Kalamata* table olives through the determination of individual phenolic compounds or the total phenolic content with the Folin–Ciocalteu assay [23,24,25,26]. To the best of our knowledge, no non-targeted screening studies have been reported to investigate the entire metabolome of *Kalamata* olives to provide information about its fingerprint. This gap could be fulfilled using the mass-spectrometric foodomics approach [12,13,27] with the development of High Resolution Mass Spectrometric (HRMS) analytical methodologies that could offer a far more comprehensive and detailed molecular picture of the composition of the *Kalamata* olives, compared to the conventional HPLC analytical methodologies [28,29,30,31]. In addition, the further coupling of non-target HRMS to chemometrics could facilitate data handling and enable the selection of characteristic markers for the *Kalamon* table olives indicative of their geographical origin. The development of chemometric geographical origin prediction models could constitute a powerful tool for the assurance of the Greek *PDO Kalamata table olives’* authenticity. In this context, the objective of this study was to develop a novel non-targeted reversed phase ultra-high pressure liquid chromatography quadrupole time-of-flight mass spectrometric method (RP-UHPLC-ESI-QTOF-MS) for the fingerprinting of *Kalamon* table olives cultivated in different regions: Kalamata (Greece), Egypt, and Chile. Partial Least Square Discriminant Analysis (PLS-DA) was used for the development of a reliable and robust prediction model able to accurately distinguish between the authentic Greek *PDO Kalamata table olives* and *Kalamata type* table olives of different origins.

## 2. Materials and Methods

### 2.1. Chemicals and Standards

All the standards and reagents were of high purity (more than 95%). Methanol (MeOH) of LC–MS grade and sodium hydroxide (purity greater than 99%) were both purchased from Merck (Darmstadt, Germany). Formic acid (LC–MS Ultra) and ammonium acetate (purity 99.0% or greater) for HPLC were purchased from Fluka (Buchs, Switzerland). Distilled water was provided by a Milli-Q purification apparatus (Direct-Q UV, Millipore, Bedford, MA, USA) and 2-propanol was purchased from Fisher Scientific (Geel, Belgium). For the validation and evaluation of the method performance, the following standard analytes were purchased and used: syringic acid (purity 95%) from Extrasynthèse (Genay, France), ferulic acid (purity 98%), gallic acid (purity 98%), epicatechin (purity 97%), p-coumaric acid (purity 98%), homovanillic acid (purity 97%), oleuropein (purity 98%), and pinoresinol (purity 95%) from Sigma-Aldrich (Steinheim, Germany), hydroxytyrosol (purity 98%) from Santa Cruz Biotechnologies and vanillin (purity 99%), caffeic acid (purity 99%) luteolin (purity 98%), apigenin (4,5,7-trihydroxyflavone; purity 97%), and tyrosol [2-(4-hydroxyphenyl) ethanol, purity 98%] from Alfa Aesar (Karlsruhe, Germany). Myricetin (purity 98%) was acquired from Sigma Aldrich and used as an internal standard (IS). Membrane syringe filters of regenerated cellulose (CHROMAFIL^®^ RC) (15-mm diameter, 0.22-μm pore size) were provided by Macherey Nagel, Düren, Germany.

### 2.2. Table Olive Samples

Sixty-five commercially available *Kalamon* table olive samples were acquired from the Greek market. The samples were all produced in the crop year 2017 and originated from Kalamata, Greece (*PDO Kalamata table olives*, 20 samples), Egypt (24 samples), and Chile (21 samples). Eleven more samples originated from Kalamata, Greece, were obtained during the following crop year (2018) and were used as a set of “suspect” samples to evaluate the prediction accuracy of the PLS-DA discrimination model. All samples had been subjected to the same de-bittering protocol involving brining with dry salt and water.

Besides the table olive samples, Quality Control (QC) samples were also prepared and analyzed throughout the batch to verify adequate analytical performance. The QC samples were prepared by mixing same-volume aliquots of all table olive extracts examined, representing both the sample matrix and the metabolic profile of the samples. Finally, four analytical procedural blanks (mixtures of MeOH: H_2_O, 80:20, *v/v*) were prepared and analyzed.

### 2.3. Sample Extraction

Firstly, the hardened endocarp (shell) was removed from the olive drupe with a sharp knife and the fleshy mesocarp (hull) and exocarp (external skin) were homogenized in a food processor and further ground into a fine paste using a mortar and pestle. Then, 0.5g (± 0.1 mg) of the homogenized sample was weighted in 2 mL Eppendorf tubes and spiked with myricetin (IS) at a final concentration of 1 mg kg^−1^. A 1 mL aliquot of MeOH: deionized water mixture (80:20, *v/v*) was used for the extraction of the analytes. The mixture was vortexed for 1 min and centrifuged for 5 min at 3400 rpm. Consequently, the upper phase was collected and filtered through RC syringe filters and 5 μL of the extract was injected into the chromatographic system.

### 2.4. UHPLC-ESI-QTOF-MS/MS Analysis

Reversed phase liquid chromatographic analysis was performed using a UHPLC system equipped with a HPG-3400 pump (Dionex UltiMate 3000 RSLC, Thermo Fisher Scientific, Dreieich, Germany) interfaced with a QTOF-MS analyzer (Maxis Impact, Bruker Daltonics, Bremen, Germany). An Acclaim RSLC C18 column (2.1 mm × 100 mm, 2.2 μm) was used for chromatographic separation, purchased from Thermo Fisher Scientific (Driesch, Germany) coupled with an ACQUITY UPLC BEH C18 precolumn (1.7 μm, VanGuard precolumn, Waters, Ireland). The column temperature was set at 30 °C. The mobile phase consisted of water: MeOH (90:10%, *v/v*) containing 5 mM ammonium acetate (solvent A), and 100% MeOH containing 5 mM ammonium acetate (solvent B). The elution gradient program adopted started with 1% of solvent B (flow rate 0.2 mL min^−1^) for 1 min, gradually increasing to 39% in the following 2 min, and then increasing to 99.9% of solvent B (flow rate 0.4 mL min^−1^) in the following 11 min. These conditions were kept constant for 2 min (flow rate 0.48 mL min^−1^), and then the initial conditions (1% solvent B, 99% solvent A) were restored within 0.1 min (flow rate decreased to 0.2 mL min^−1^) to re-equilibrate the column for 3 min before the next injection.

The QTOF MS system was equipped with an electrospray ionization (ESI) interface, operating in negative mode with capillary voltage of 3500 V, end plate offset of 500 V, nebulizer pressure of 2 bar (N_2_), drying gas flow rate of 8 L min^−1^ (N_2_), and drying temperature of 200 °C. A QTOF external calibration was performed daily using a sodium formate cluster solution, and an internal calibration was performed in every chromatogram, using a calibrant injection at the beginning of each run (0.1−0.25 min). The sodium formate calibration mixture consisted of 10 mM sodium formate in a mixture of water and 2-propanol (1:1, *v/v*). Full scan mass spectra were recorded in the range from 50 to 1000 *m*/*z*, with a scan rate of 2 Hz. MS/MS experiments were conducted using data dependent AutoMS mode in which the five ions with the highest abundance per MS scan are fragmented with a pre-defined collision energy set based on the mass and charge stante of the ions. AutoMS mode provides clear and compound-specific MS/MS spectra, enabling the structure elucidation of unknown compounds. The analytical instrument provided a typical resolving power (full width at half maximum) between 36,000 and 40,000 at *m*/*z* 226.1593, 430.9137, and 702.8636.

### 2.5. Method Performance Evaluation

Τhe performance of the UHPLC-ESI-QTOF-MS/MS methodology was evaluated in order to verify its performance characteristics. A set of 14 phenolic compounds was used for the assessment of the applicability of the methodology and several performance characteristics were examined such as the method’s linearity, accuracy, precision, sensitivity, and matrix effects. A target list was created, including the phenolic compounds that belong to the classes of phenolic acids, flavonoids secoiridoids, and lignans, and that have already been identified in Greek table olives and olive oils [23,25]. The compiled target list is presented in Table S1 in the Electronic Supplementary Material, including the names and molecular formulas of the compounds, as well as their molecular ions in negative ionization mode (M − H)^−^, retention times (t_R_), and most abundant fragment ions. The data processing was performed using Bruker Daltonics Data Analysis 4.1 and TASQ Client 1.4 software packages (Bremen, Germany).

Standard addition curves were constructed to assess the linearity of the method. Caffeic acid, ferulic acid, gallic acid, syringic acid, p-coumaric acid, homovanillic acid, pinoresinol, apigenin, vanillin, epicatechin, luteolin, hydroxytyrosol, tyrosol, and oleuropein were spiked at concentrations between 0.02 and 10 mg·kg^−1^ (10 calibration levels with 3 replicates at each level). Myricetin, a phenolic compound that did not exist in the matrix, was chosen and used as an IS to compensate for recovery losses and to correct any potential drift of the analytical signal throughout the batch. The calibration curves were constructed by correlating the spiked concentration of each analyte to the peak area of the spiked analyte, after subtracting the peak area of the neat sample, and divided by the peak area of the IS. The limits of detection (LODs) and limits of quantification (LOQs) of the method were evaluated using the standard error and slopes of low-concentration standard addition calibration curves, constructed using the lowest concentration range 0.02–1 mg kg^−1^, according to the following equations [5]:

(1) $$LOD=\frac{3.3\times S_{a}}{b}$$

(2) $$LOQ=\frac{10\times S_{a}}{b}$$

where *S_a_* is the standard error of the intercept *a* and *b* is the slope.

The accuracy of the method was estimated by calculating the relative recovery of all target analytes at the 0.5 mg·kg^−1^ concentration level. The matrix effect was also calculated at the 0.5 mg·kg^−1^ concentration level and, finally, the precision of the method was estimated as repeatability (intra-day precision) and intra-laboratory reproducibility (inter-day precision). Repeatability was expressed as the %RSDr values of eight replicate analyses (*n* = 8) of spiked samples at a concentration of 0.5 mg kg^−1^ analyzed at the same laboratory day, while the reproducibility results were expressed as the%RSDR value of three replicates of spiked table olive samples analyzed during two consecutive days (*n* × k = 3 × 2 = 6).

### 2.6. Non-Target Screening Methodology

#### 2.6.1. Data Processing

The raw data from the analysis of 65 samples were converted into mzXML files using the MSconvert module from Proteowizard [32]. These raw data were then imported into R workspace and the centWave algorithm implemented in XCMS R package was used to perform peak picking [33]. The internal parameters of XCMS were optimized by IPO R package using the analyzed QC samples. The optimized values of ppm (threshold for tolerated mass deviation), minimum and maximum chromatographic peak width were 23.3, 17.5, and 40, respectively. The signal-to-noise and the intensity threshold was set to 3 and 1000. The “prefilter” parameter, being the threshold for an *m*/*z* to be considered as a true peak if it exists in k consecutive scans at J intensity threshold (k, I), was set at 3–1000. The rector and group functions were used from XCMS to align the retention time data and group the detected peaks across all samples. The retention time alignment method was based on the non-linear retention time alignment wrapping algorithm (loess). The annotation of extracted *m*/*z* features was done by CAMERA and non-target R package. This was to prevent adducts/isotopic peaks to cofound with their molecular ions. The masses (*m*/*z*) present within the analytical procedural blank were removed from sample peaks-list using an advanced chemometric method based on deep learning [34]. Furthermore, the (MS-FLO) approach (https://msflo.fiehnlab.ucdavis.edu/#/) was used to improve the quality of the feature lists after initial processing, for instance by removing the duplicates and decreasing the initial list of 4144 *m*/*z* features to 2108. Eventually, a table including 65 samples and 2108 *m*/*z* features was generated. Each of these *m*/*z* values could be a potential mass of interest to be identified.

Consequently, a normalization or transformation of the data was necessary before performing the chemometric data analysis on the peaks-list, [35,36,37,38]. Normalization omits the influence of confounding variations that might have been comprised from experimental sources, such as experimental bias, analytical noise, or instrumental sensitivity. Moreover, the signal of a compound with lower concentration can be easily manipulated by the analytical noise, and scaling of the peaks-list is a necessity in order to compare different biomarkers. Here, the optimal division of the peak-list consisted of 65 samples and 2108 *m*/*z* values into training (51 samples) and independent test (14 samples); the subset was achieved based on the Kennard–Stone approach [39]. The Kennard-Stone dataset division method begins by selecting a pair of points (i samples and *m*/*z* features) that are the furthest apart. The selected samples are assigned to the training sets and then excluded from the initial list. Then, the next pair of samples, which are the second furthest apart, are listed into the test subset. The procedure continues to assign each remaining sample alternatively to the training and test sets based on a distance function and compared to the previously selected samples. The distance function used was Euclidean distance. Prior to the final discrimination model, the peaks-list (training and test subset) was scaled. In this case, Pareto scaling was selected after evaluating various pretreatment methods including range scaling, Mean centering, Autoscaling, variable stability (Vast) scaling [40], Level scaling [35], Variance Stabilizing Normalization (VSN) [41], mean centered log2 transformation [42], and Probabilistic Quotient Normalization (PQN) [43]. PQN is based on calculating the most probable dilution factor by looking at the distribution of the quotients of the response factor of a test spectrum by those of a reference spectrum. For calculating the quotients in the PQN method, a reference spectrum was needed. The reference spectrum could be a single representative spectrum of the study, or a calculated median or mean spectrum of a subset of controls samples (QC). When using PQN, the median as an estimation of the most probable quotient was applied on the set of 6 QC samples.

#### 2.6.2. Chemometrics

Chemometrics-based classification methods were used to discriminate the Greek, Egyptian, and Chilean *Kalamon* olives based on their geographical origins and to reveal characteristic geographical origin markers. At first, Principal Component Analysis (PCA) was used to perform data exploratory analysis for QC and analytical procedural blank samples. Consequently, the peaks-list generated from the UHPLC-QTOF-MS analysis of the samples was used for PLS-DA [44]. More details about PLS-DA can be found elsewhere [45]. The optimal number of 3 latent variables (LVs) was selected after evaluating the miss-classification error in 5-leave out cross validation. Variable Importance in Projection (VIP) [46] was also used to find the potential markers revealed by the PLS-DA model discriminating between Greek, Egyptian, and Chilean table olive samples. VIP scores represent the influence of each individual *m*/*z* value and they are calculated as the weighted sum of squares of the PLS-DAs (PLS-DA1, PLS-DA2 etc.) weights. The cut-off value of above 0.83 was used for the VIP score [47]. In addition, in order to highlight the key features most responsible for the differentiation of the *Kalamon* olives, an orthogonal PLS-DA (OPLS-DA) was employed. One class was faced against two other ones in turn, so that the pairwise VIP values could be derived. To build the aforementioned supervised method and conduct cross-validation analysis, the PLS-DA toolbox was written in MATLAB [45] and R (*ropls* R package) [48] were used.

#### 2.6.3. Model Validation and Classification Performance

The PLS-DA model was validated internally and externally using mis-classification error rate in leave- many-out cross validation and using 14 table olive samples as the test set. The total error rate, class specificity, and sensitivity, as well as class assigned probabilities for training, k-fold-cross-validation (k = 5), and test set were calculated and used in Receiver Operating Characteristics (ROC) curve. Considering these validation steps, a classification model is internally appropriate when it gives a point in the upper left corner of the ROC curve, providing the total area under curve equal to 1 as well as the maximum sensitivity and specificity. However, a random model gives a curve positioned around diagonal line from the left bottom to the top right corner [45]. Several statistical parameters were calculated for ranking and evaluating the PLS-DA model. These parameters were R^2^X (measures the accumulative variance explained by selected number of latent variables), R^2^Y (measures the goodness of fit), Q^2^ (k-fold-cross-validated, predictive ability of the model), as well as RMSEE (Root Mean Square Error of Estimation) and RMSEP (Root Mean Square Error of Prediction), which are the square root of the mean error between the actual and the predicted classes for the training and test set, respectively. The statistical significance of the PLS-DA model was also assessed by the diagnostic statistics of Q residuals and Hotelling’s T^2^ (for outlier analysis) [45] and the permutation test of cumulative R^2^Y and Q^2^ values [49]. Hotelling’s T^2^ test is based on the sum of normalized squared scores, which calculates the variation for each sample within the developed supervised classification model. A high Hotelling’s T^2^ value means that the sample is distant from the center of the established model. On the other hand, a high Q residual indicates that there is a high difference between the actual data and the one that is reconstructed from the supervised classification model. Therefore, any samples with higher Hotelling’s T^2^ and Q residual values can be considered as outliers.

#### 2.6.4. Marker Identification

Variable Importance in Projection (VIP) [46] values revealed all the significant markers that were responsible for the discrimination between the Greek, Egyptian, and Chilean *Kalamon* table olive samples. These markers were identified using an in-house compound identification protocol and following the identification confidence levels for HRMS proposed by Schymanski et al. [50].

At first, the corresponding *m*/*z* features (reaching to the level of identification 5) were looked up within a detailed list of known unknowns including 259,298 natural products, built by our group, as well as PubChem. The look up task was done with a mass accuracy threshold of below 5 mDa. Next, the theoretical isotopic pattern was calculated for each matched candidate by “enviPat” R package [51] and then compared with the observed experimental isotopic pattern using dot product algorithm (reaching to the level of identification 4). Isotopic fit was expressed as mSigma with a threshold for positive identification of < 100. Then, the experimental and predicted retention time of each candidate was evaluated to exclude any false positives. For the interpretation of the MS/MS fragments as well as ranking (based on their explained MS/MS fragments and mass accuracy), the in silico fragmentation tools of MetFrag [52] and CFM-ID [53] were used (reaching to the level of identification confidence 3). The online public mass spectrum libraries (mzcloud, massBank, METLIN, and GNPS) were also used to increase the identification confidence of the compounds to 2a. In this regard, a MS/MS similarity score threshold of 0.7 was used between the MS/MS spectra of the candidates and the spectra of reference standards reported. Where no intensity information was available for the MS/MS spectra of reference standard, the presence of at least three matched fragments was required to positive identify the detected compound. All of these steps were done using an in-house program called “AutoSuspect” [54].

## 3. Results and Discussion

### 3.1. Method Performance Results

The calibration curves were constructed, and excellent linearity was achieved (r^2^ > 0.99, in all cases). The precision limit was ≤ 3.6% RSD for intra-day experiments and ≤ 4.4% RSD for inter-day experiments, demonstrating the good precision of the method developed. LODs and LOQs were satisfactory and ranged between 0.019 (apigenin) and 0.041 (*p*-coumaric acid) mg kg^−1^ and 0.056 (apigenin) and 0.091 (gallic acid) mg kg^−1^, respectively. All examined analytes showed adequate recovery efficiency (between 81.4% and 94%) and low matrix suppression (ME%: 8.09–19.4). The method performance parameters of the developed methodology are presented in Table 1.

### 3.2. Non-Target Screening Results

#### 3.2.1. Data Processing

Nine different scaling methods were tested, and Pareto scaling was selected as the optimum after evaluating the R^2^X, R^2^Y, Q^2^, and ROC curve, as well as RMSEE and RMSEP. Table S2 in the Electronic Supplementary Material shows the evaluation results of each scaling method tested. Most of the scaling methods showed comparable R^2^Y and Q^2^ values (except for VSN) whereas Pareto scaling showed all in all satisfactory results (with the score of 0.932) for RMSEP and RMSEE. PQN, tested for the first time in authenticity studies of olive drupes by HRMS, provided relatively compatible statistical results in comparison with other methods (Pareto scaling, mean centering, and Level). PQN is a robust method to reduce any variance arising from subtly differing dilutions between the extracts. Although PQN was not used for the final scaling method after considering the final score, it can have a good potential use in foodomics studies. The score was developed based on the importance of each statistical parameter to rank the scaling methods used. Equation (3) shows the weights used for each parameter:

(3) $$\begin{array}{cc} Score=0.15\left(R^{2}X\right) & +0.15\left(R^{2}Y\right)+0.20\left(Q^{2}\right)+0.15\left(1-\mathrm{RMSEE}\right)\\ & +0.30\left(1-\mathrm{RMSEP}\right)+0.01\left({\mathrm{ACC}\;}_{\mathrm{Train}}\right)+0.02\left({\mathrm{ACC}\;}_{\mathrm{Test}}\right)\\ & +0.02\left({\mathrm{ACC}\;}_{\mathrm{cross}\;\mathrm{validation}}\right) \end{array}$$

#### 3.2.2. Chemometrics

**Figure 1:**
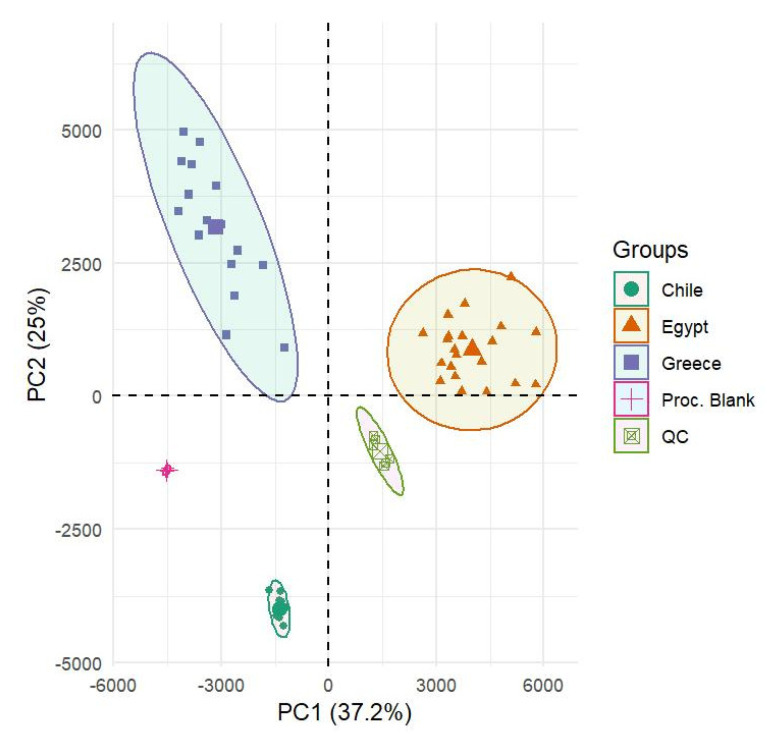
Principal Component Analysis (PCA) score plot of Quality Control (QC) samples, analytical procedural blank, and *Kalamon* table olives from different geographical origin.

PCA was used in order to qualitatively evaluate the distribution of the samples on the score plot before establishing the final supervised classification model. As depicted in Figure 1, PCA was applied on the scaled peaks-list including 2108 *m*/*z* values and 65 samples, as well as 6 QCs and 4 analytical procedural blanks. This was important to verify that the analytical procedural blank and QC samples were grouped together and apart from the other samples. A clear separation was also observed between samples (within 95% confidence intervals) from the different geographical origins explaining over 62% of variance.

**Figure 2:**
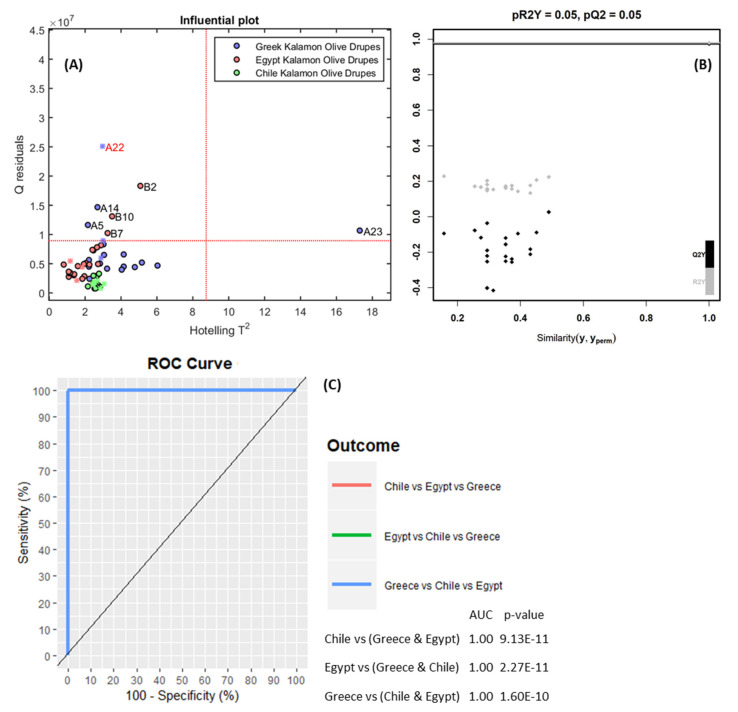
(**A**) Influential analysis by Hotelling’s T^2^ distribution; (**B**) Permutation test of cumulative R^2^Y and Q^2^ values; (**C**) Receiver Operating Characteristics (ROC) curve for the developed PLS-DA.

Consequently, a PLS-DA supervised classification model was developed using the scaled peaks-list from Pareto scaling and 51 out of 65 samples as the training set. The remaining 14 samples were used as a test set to externally evaluate the accuracy of the PLS-DA model. Three latent variables were selected for PLS-DA according to the leave-k-out cross validation (k = 5) results. The mis-classification error rate for leave-one-out cross validation, the test set, and the training set was 0.00. The plot of Hotelling’s T^2^ versus Q residuals was constructed, and is shown in Figure 2A. All classes had only a few samples with high Q residuals and only one sample (“A23”) higher than the confidence limits (both Hotelling’s T^2^ value and Q residuals). However, attempting to exclude this sample did not affect the overall classification performance and PLS-DA model; therefore, the “A23” sample was not excluded from the dataset. After 20 random permutations of the classes, the random PLS-DA had a significantly lower Q^2^Y value than the one obtained by the trained PLS-DA model, as shown in Figure 2B. This confirms that overfitting did not occur. ROC curves were also calculated for each class (plots of sensitivity versus 100-specificity) and the area under curve was calculated as 1.00, representing an excellent classification model (Figure 2C).

**Figure 3:**
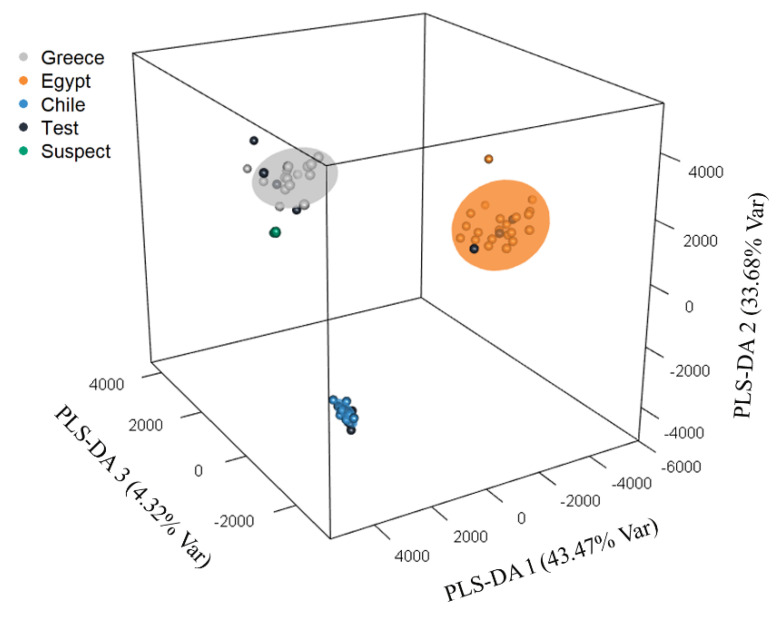
PLS-DA model for the discrimination of *Kalamon* olive drupes of different geographical origins.

Finally, the developed PLS-DA model’s robustness was proven by analyzing a set of “suspect” samples, consisting of 11 table olive samples of the Greek *Kalamata* olives collected in a different harvesting year (2018). Figure 3 shows the final PLS-DA model, along with the test set and suspect samples located within the 95% confidence intervals of each geographical origin. Even though different environmental conditions could cause variation in the chemical content of table olive samples from different years, the suspect samples were grouped very closely to the training set used by the PLS-DA model and were classified correctly as Greek *Kalamon* olive drupes. This proved the robustness of the model and its applicability to the accurate discrimination of Greek, Egyptian, and Chilean *Kalamon* olive drupes, indicating the potential use of the current workflow for PDO authentication purposes.

#### 3.2.3. Marker Identification

With the use of 65 samples and a peak-list of 2108 features (*m*/*z*), PLS-DA (from VIP values) indicated 26 *m*/*z* features that are significant to discriminate the geographical origins of the *Kalamon* table olive samples (Greek, Chilean, or Egyptian). The list of these *m*/*z* features can be found in Table 2.

**Figure 4:**
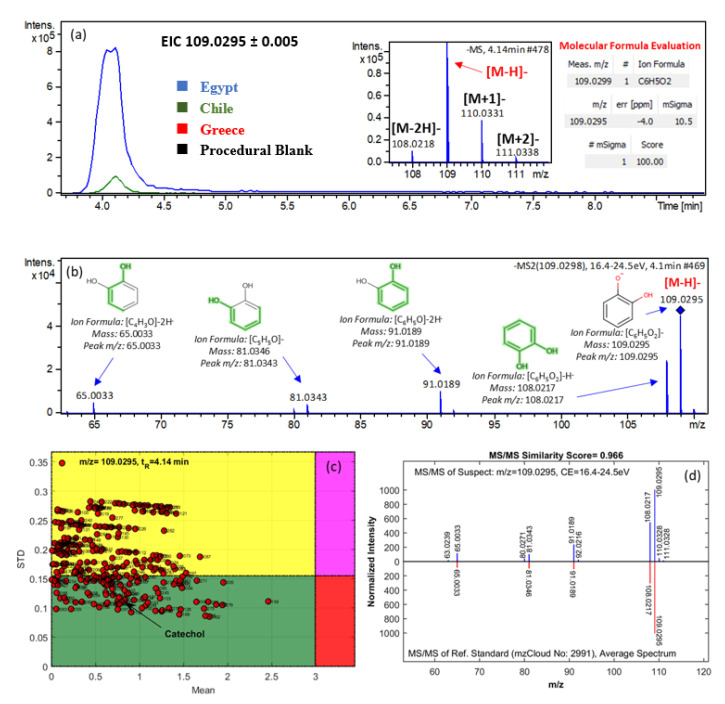
Identification of **catechol**: (**a**) fullscan Mass Spectrometry (MS) chromatogram and Extracted Ion Chromatogram (EIC) for the given mass (±5 mDa); (**b**) MS/MS spectra and corresponding fragments; (**c**) Monte Carlo Sampling (MCS) plot for evaluating the predicted t_R_ values; (**d**) confirmation step using MS2 spectra of reference standard from the spectrum library.

The identification methodology that was used is described in Section 2.6.4. One of the most important markers, detected in high abundance in Egyptian *Kalamata type* table olives, was the mass feature with *m*/*z* 109.0299 and t_R_ = 4.14 min (Figure 4). For this mass feature, the molecular formula C_6_H_6_O_2_ was assigned using Bruker “Smart Formula Manually” with mass accuracy and isotopic fit (mSigma) being −0.4 mDa and 10.5, respectively (Figure 4a). Then, 371 compounds were retrieved from PubChem using a mass accuracy threshold of 5 ppm. Afterwards, the candidates were processed by MetFrag [52] and an in-house QSRR-based retention time prediction model that was developed by our group [55]. The most probable candidate was found to be catechol and the elucidated chemical structures of the fragments are given in Figure 4b. According to the degree of MEAN value (absolute values of mean of predictive residuals) in the Monte Carlo Sampling (MCS) plot [55], this candidate was not classified as a potential false positive (Figure 4c). The spectra of the reference standard of catechol was found in mzCloud database (mzCloud No: 2991) and the fragments matched the ones of the mass feature detected (Figure 4d). Therefore, the identification was confirmed by t_R_ prediction, MCS plot, and MS/MS comparison (spectra similarity score of 0.966) at a level of identification confidence of 2a.

Two mass features with the same retention time were indicated from the PLS-DA model to be important for the Greek, Chilean, and Egyptian *Kalamon* olive discrimination: the marker detected at *m*/*z* 153.0556_t_R_ = 3.53 min, as well as the marker detected at *m*/*z* 123.0452_t_R_ = 3.53 min. For the first compound, the molecular formula of C_8_H_10_O_3_ was assigned (mass accuracy: 0.1mDa, mSigma 42.6) and after evaluation of MS/MS fragments and retention time, it was found to be hydroxytyrosol, for which a reference standard was also available (identification level 1). For the marker detected at *m*/*z* 123.0452_t_R_ = 3.53 min, the molecular formula C_7_H_8_O_2_ was assigned using “SmartFormula Manually”, fulfilling the required criteria of mass accuracy (−0.1 mDa) and isotopic fit (9.4 mSigma). This *m*/*z* was also found to be present in the MS and MS/MS spectrum of hydroxytyrosol (Figure S1b,c in the Electronic Supplementary Material). Therefore, it was concluded that *m*/*z* 123.0447_t_R_ = 3.53 min is an in-source fragment of hydroxytyrosol with its most possible and appropriate structure being methyl-catechol moiety.

For the marker detected at *m*/*z* 137.0609_t_R_ = 4.14 min, the molecular formula of C_8_H_10_O_2_ was suggested (mass accuracy: −0.1mDa, mSigma 19.2). After the evaluation of the MS/MS fragments and retention time it was confirmed that the compound was tyrosol, which had also been used for the method performance evaluation (level of identification 1).

For the mass feature detected at *m*/*z* 133.0137_t_R_ = 1.15 min, which was more abundant in the Greek olives than in Egyptian and Chilean ones, the molecular formula of C_4_H_6_O_5_ was assigned with a mass accuracy and isotopic fit of 0.5 mDa and 10.6 mSigma, respectively. Sixty-two candidates were retrieved from PubChem and processed using MetFrag. This list was further filtered with the use of the QSRR-based retention time prediction model. Twelve potential candidates presented a MetFrag Score above 0.5 and retention time error below 1.8 min, among which L-malic acid for which a reference standard was available in our lab. After the analysis of the reference standard, the mass feature with *m*/*z* 133.0137_t_R_ = 1.15 min was positively identified as L-malic acid. Another marker compound detected in high abundance in Greek olive drupes was *m*/*z* 191.0562_t_R_ = 1.60 min, with an assigned molecular formula of C_7_H_12_O_6_ (mass accuracy 0.1mDa, mSigma 18.6). Then, 702 candidates were retrieved from PubChem and evaluated by MetFrag score and MCS value. This *m*/*z* marker was identified as quinic acid with a level of identification 2a after matching the MS/MS fragments with those in the METLIN database (METLIN ID: 3329).

Several mass features were detected in high abundance in Chilean olives, such as *m*/*z* 269.0453_t_R_ = 8.23, *m*/*z* 285.0403_t_R_ = 8.20, and *m*/*z* 285.0403_t_R_ = 7.51 min. These markers were identified as the flavonoids apigenin, kaempferol, and luteolin, respectively, after evaluating the mass accuracy, isotopic pattern, experimental retention time, and MS/MS match with reference standards.

The next group of markers, identified with levels of confidence 1 and 2a, belong to the fatty acids group and were found at high levels in Egyptian and Greek *Kalamon* table olives. Alpha-Linolenic acid (*m*/*z* 277.2172_t_R_ = 12.73 min, C_18_H_30_O_2_) and linoleic acid (*m*/*z* 279.2329_t_R_ = 13.23 min, C_18_H_32_O_2_) were found to be characteristic markers for Egyptian olives, while **oleic acid** (*m*/*z* 281.2488_t_R_ = 13.77 min, C_18_H_34_O_2_), 13-Keto-octadeca-9*Z*,11*E*-dienoic acid (*m*/*z* 293.2120_t_R_ = 10.99 min, C_18_H_30_O_3_, reference standard MS/MS spectra available at METLIN id 34474), 9*R*-hydroxy-10*E*,12*Z*-octadecadienoic acid (*m*/*z* 295.2276_t_R_ =11.10, C_18_H_32_O_3_, reference standard MS/MS spectra available at METLIN id 45660), trans-EKODE-(E)-Ib (*m*/*z* 309.2069_t_R_ = 9.35 min, C_18_H_30_O_4_, reference standard MS/MS spectra available at METLIN id 35761), and **(±)9-HpODE** (*m*/*z* 311.2224_t_R_ = 9.84 min, C_18_H_32_O_4_, reference standard MS/MS spectra available at METLIN id 64785) were established as characteristic markers of *PDO Greek Kalamata table olives*.

**Figure 5:**
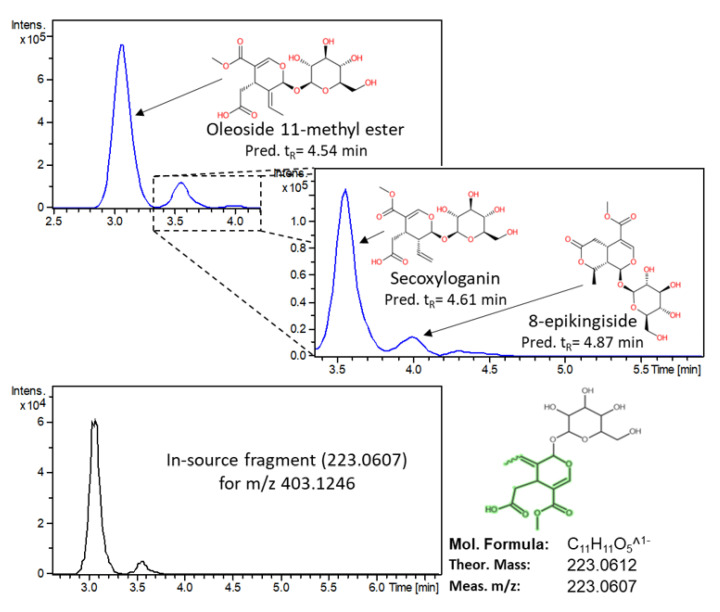
Structural elucidation and prioritization of candidates for the *m*/*z* value 403.1246.

Three isobaric compounds were determined with *m*/*z* 403.1244 and t_R_ = 3.08, 3.58, 4.03 min and were indicated by the PLS-DA model to play an important role in the geographical origin determination of the *Kalamon* olives. For these compounds, the C_17_H_24_O_11_ molecular formula was assigned with a mass accuracy and an isotopic fit of 0.2mDa and 25.6 mSigma, respectively. After the evaluation of MS/MS fragmentation and retention time, several tentative candidates were found in the literature [56]. Therefore, three secoiridoids, namely Oleoside methyl ester, 8-epikingiside, and secoxyloganin were tentatively identified at a level of identification 3. Since they all share the common fragment of 165.0554 (loss of glycoside and methyl ester moiety) and 223.0607 (loss of glycoside moiety), it was difficult to distinguish between these candidates based on MS/MS fragmentation and, therefore, the predicted retention time was used to prioritize them (as shown in Figure 5). In addition, the in-source fragment of 223.0607 was detected in the MS spectrum.

**Figure 6:**
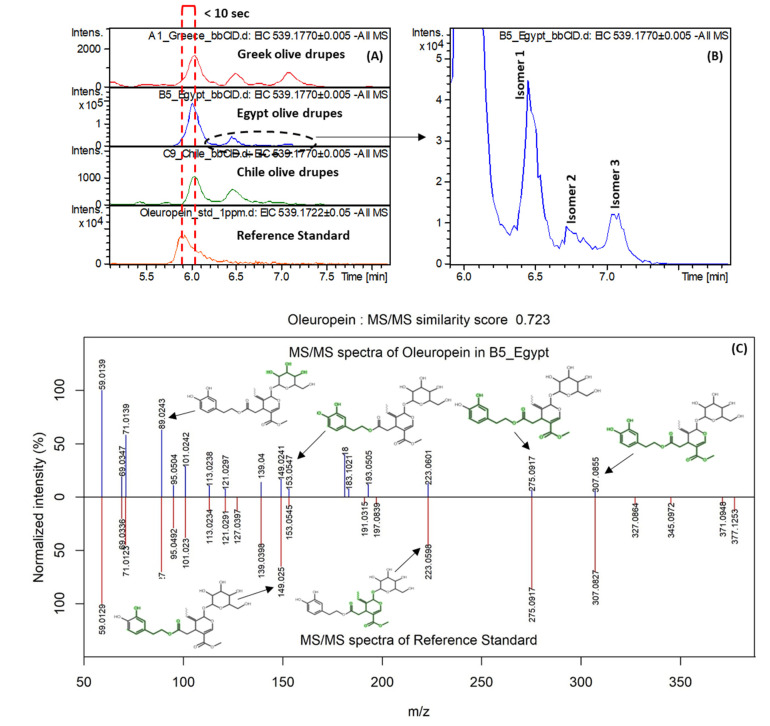
(**A**) EIC of oleuropein for representative samples from each geographical origin and reference standard; (**B**) isomers of oleuropein detected in olive drupes; (**C**) MS/MS match between oleuropein in the samples and its reference standard.

Apart from the above mentioned secoiridoids, three additional compounds from this class including *p*-coumaroyl-6-secologanoside [6], oleuropein, and dihydrooleuropein were identified at the level of identification of 2a, 1, and 3, respectively. *p*-Coumaroyl-6-secologanoside was assigned to the *m*/*z* feature 535.1455_t_R_ = 4.61 min with a molecular formula C_25_H_28_O_13_ (mass accuracy 0.2 mDa and mSigma 11.1). The predicted retention time and MCS value was in line with the experimental. Two characteristic MS/MS fragments were reported in the literature [56] for *p*-coumaroyl-6-secologanoside (*m*/*z* 265.0728 and 491.1560), matching to the experimental MS/MS spectra (265.0715 (S/N = 15.8, ionic formula = C_13_H_13_O_6_, 0.3 mDa) and 491.1547 (S/N = 2.5, ionic formula = C_24_H_27_O_11_, 1.2 mDa) of the samples from Greek *Kalamon* variety. The next important marker (*m*/*z* 539.1764_t_R_ = 6.47 min), highly abundant in Egyptian *Kalamata type* table olives, was identified as oleuropein. The mass accuracy and isotopic fitting were 0.6 mDa and 93.2 mSigma, respectively. After the retention time and MS/MS evaluation, the most probable candidate was oleuropein and it was positively identified using the corresponding reference standard (level of identification 1). Three more isomers of oleuropein were observed at retention times of 6.46, 6.72, and 7.05 min. These isomers have been also reported previously [56]. Figure 6 shows the matched information between oleuropein and the reference standard as well as observed isomers.

**Figure 7:**
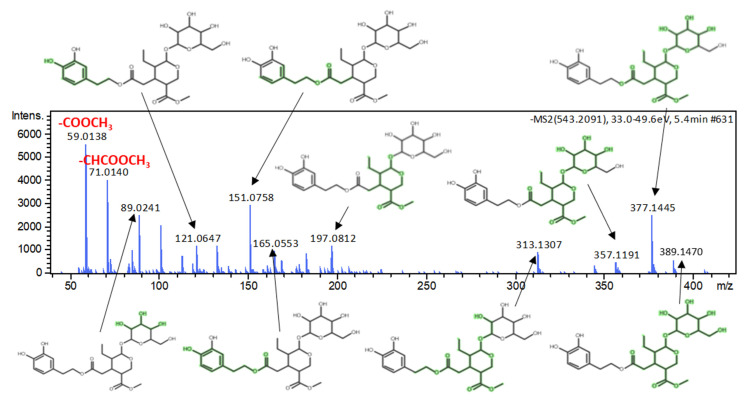
The structural elucidation of MS/MS fragments of dihydrooleuropein.

Dihydrooleuropein (molecular formula of C_25_H_36_O_13_, mass accuracy of 0.3 mDa, 23.0 mSigma) was tentatively assigned to the mass feature with *m*/*z* 543.2080_t_R_ = 5.39 min. The prediction of retention time for this compound failed due to the fact that it lies outside of the application domain of the model in ESI negative mode. Therefore, this candidate was tentatively identified after evaluating its MS/MS fragments with the in silico fragmentation tool MetFrag (Figure 7).

Three more characteristic markers of Egyptian *Kalamon* table olives were tentatively identified as Verbascoside, Isoacteoside, and Campneoside II, belonging to the class of phenylpropanoids. These compounds give a diagnostic fragment ion at *m*/*z* 461.1670 that relates to the caffeoyl moiety loss [56]. Verbascoside and Isoacteoside are isomeric compounds and give similar MS/MS spectra. Unfortunately, we could not use the retention time prediction model to define their elution order as these compounds are out of the application domain of the model

**Figure 8:**
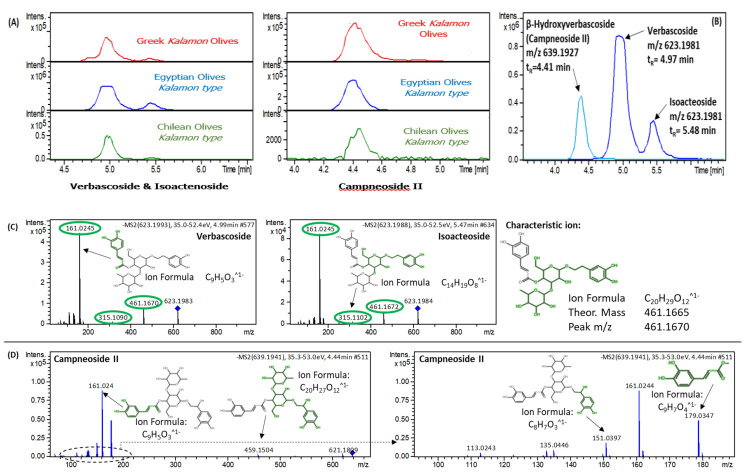
(**A**) abundance and (**B**) elution order of all phenylpropanoids in Greek, Egyptian, and Chilean table olive samples; (**C**) diagnostic fragment loss of caffeoyl moiety in Verbascoside and Isoacteoside; (**D**) MS/MS evaluation of Campneoside II.

For the third mass feature with *m*/*z* 639.1927, only one peak was detected representing the hydroxyl form of one of these isomers (C_29_H_36_O_16_ with isotopic pattern fit of 20.5 mSigma and mass accuracy of 0.4 mDa). According to the literature, β-Hydroxyverbascoside (Campneoside II) exists dominantly in olive drupes [56]. Therefore, evaluating the polarity and similarity of these three compounds, we can assume that Verbascoside would elute closer to β-Hydroxyverbascoside than Isoacteoside and, thus, the isomer eluting at 4.97 min is Verbascoside. Figure 8 shows the abundance of these phenylpropanoid compounds in all samples, the assigned compounds based on elution order, and their MS/MS evaluation.

The molecular formula of C_9_H_8_O_4_ (with the mass accuracy of −0.2 and isotopic fit of 16.1) was assigned on the mass detected at *m*/*z* 179.0350, t_R_ = 4.16 min. We retrieved 810 candidates from PubChem using the assigned molecular formula. After evaluating the MetFrag score and MCS value, caffeic acid and some derivatives of cinnamic acid as well as coumaric acid were found to be in the top hit list. However, caffeic acid was rejected as a potential candidate due to the retention time shift in comparison with the available reference standard. Although this peak shares common fragments (123.0442 and 137.0238) in contrast to hydroxytyrosol and cinnamic acid derivatives which could reveal the substructure, it could also be the in-source fragment of the *m*/*z* 639.1927, t_R_ = 4.41 min. Therefore, the level of identification 4 was assigned to this *m*/*z* due to the lack of extra evidence for further prioritization.

## 4. Conclusions

This study examined the differences between the metabolomic fingerprints of *Kalamon* table olives of different geographical origins (Greece, Egypt, and Chile). A novel non-targeted UHPLC-ESI-QTOF-MS/MS methodology was developed and evaluated, providing adequate analytical performance characteristics. The non-target results were used for the development of a PLS-DA prediction model that successfully discriminated the *PDO Greek Kalamata table olives* from *Kalamata type table olives* from Egypt and Chile. Twenty-six compounds were suggested as characteristic authenticity markers responsible for the discrimination between the samples and all of them were tentatively identified using an in-house marker identification workflow. This study provides a comprehensive workflow that can be used to guarantee PDO product authenticity preventing fraud, protecting the consumers, and stopping unfair competition between producers.

## Figures and Tables

**Table 1:** Reversed phase ultra-high-pressure liquid chromatography quadrupole time-of-flight mass spectrometric (RP-UHPLC-ESI-QTOF-MS/MS) method performance parameters.

Compound	LOD (mg kg^−1^)	LOQ (mg kg^−1^)	Intra-day Precision RSD_r_% (*n* = 3)	Inter-day Precision RSD_R_% (*n* = 3 × 2)	Equation y = (a ± Sa) + (b ± Sb)x Linear Range: 0.02–10 mg kg^−1^	r^2^	RE%	ME%
Gallic acid	0.035	0.098	4.5	5.2	y = (0.04 ± 0.03) + (0.11 ± 0.09)x	0.991	93.4	−10.2
*p*-Coumaric acid	0.041	0.096	5.4	6.2	y = (0.03 ± 0.02) + (0.04 ± 0.01)x	0.991	87.1	−11.8
Ferulic acid	0.039	0.089	5.7	6.4	y = (0.05 ± 0.04) + (0.037 ± 0.006)x	0.994	83.2	−15.9
Syringic acid	0.032	0.092	6.4	6.8	y = (0.007 ± 0.002) + (0.07 ± 0.01)x	0.992	92.4	−17.4
Homovanillic acid	0.034	0.085	4.2	6.1	y = (0.005 ± 0.002) + (0.02 ± 0.01)x	0.995	90.4	−12.9
Tyrosol	0.035	0.094	4.8	5.9	y = (−0.006 ± 0.003) + (0.031 ± 0.005)x	0.997	92.4	−9.75
Hydroxytyrosol	0.038	0.079	3.9	5.1	y = (−0.027 ± 0.004) + (0.09 ± 0.06)x	0.994	91.5	−11.1
Pinoresinol	0.021	0.078	4.1	4.4	y = (−0.009 ± 0.004) + (0.03 ± 0.01)x	0.993	95.1	−8.09
Apigenin	0.019	0.056	4.8	5.1	y = (0.10 ± 0.07) + (0.25 ± 0.37)x	0.992	89.7	−18.8
Oleuropein	0.027	0.074	3.6	4.9	y = (−0.019 ± 0.002) + (0.13 ± 0.02)x	0.994	90.1	−13.7
Vanillin	0.034	0.076	4.7	6.1	y = (−0.02 ± 0.01) + (0.19 ± 0.07)x	0.996	86.7	−16.5
Caffeic acid	0.029	0.088	5.6	6.3	y = (−0.03 ± 0.01) + (0.171 ± 0.004)x	0.993	82.5	−15.2
Epicatechin	0.036	0.082	4.8	5.5	y = (−0.02 ± 0.01) + (0.046 ± 0.007)x	0.992	81.4	−19.4
Luteolin	0.023	0.069	3.9	4.4	y = (−0.015 ± 0.001)x + (0.03 ± 0.02)x	0.991	94.0	−9.41

**Table 2:** List of the markers detected in olive drupes from different geographical origins.

Compound Name	Class	Theoretical *m*/*z* [M − H]^−^	Molecular Formula	Experimental t_R_ (Predicted t_R_) min	Characteristic Marker of	Pairwise VIP Values (from PLS-DA)			MS/MS Fragments (the 3–5 Most Abundant Fragments) in RPLC-(-ESI)-QTOF-MS	Level of Identification Confidence
						Greece vs. Egypt	Greece vs. Chile	Egypt vs. Chile		
Catechol	Phenols	109.0295	C_6_H_6_O_2_	4.14 (5.09)	Egypt	9.916	1.394	10.202	65.0033, 81.0346, 91.0189, 108.0217, 109.0295	2a
Hydroxytyrosol	Phenols	153.0557	C_8_H_10_O_3_	3.53	Egypt	13.751	13.459	1.729	81.0347, 95.0504, 108.0217, 123.0453, 137.0242	1
Methyl-catechol (in-source fragment of Hydroxytyrosol)	Phenols	123.0452	C_7_H_8_O_2_	3.53	Egypt	13.357	9.357	6.027	67.0191, 95.0503, 123.0451	4
Tyrosol	Phenols	137.0608	C_8_H_10_O_2_	4.14	Egypt	1.47	2.441	2.347	106.0417, 119.0502, 137.0608	1
L-(−)-Malic acid	Carboxylic Acids	133.0142	C_4_H_6_O_5_	1.15	Greece	5.397	5.927	0.197	59.0139, 71.0139, 72.9931, 89.0243, 115.0034	1
Quinic acid	Alcohols & polyols	191.0561	C7H12O6	1.60 (1.41)	Greece	1.557	4.431	3.047	85.0292, 87.0086, 93.0343, 127.0394, 191.0550	2a
Apigenin	Flavonoids	269.0455	C_15_H_10_O_5_	8.23	Chile	0.098	2.504	2.724	65.0031, 117.0342, 149.0238, 151.0027, 225.0542	1
Kaempferol	Flavonoids	285.0405	C_15_H_10_O_6_	8.20	Chile	2.171	6.419	5.642	65.0032, 133.0290, 151.0031, 175.0395, 199.0393	1
Luteolin	Flavonoids	285.0405	C_15_H_10_O_6_	7.51	Chile	2.41	7.174	6.202	65.0032, 133.0290, 151.0031, 175.0395, 199.0393	1
alpha-Linolenic acid	Fatty acids	277.2173	C_18_H_30_O_2_	12.73	Egypt	0.368	2.018	1.88	277.2168, 278.2204	1
Linoleic acid	Fatty acids	279.2330	C_18_H_32_O_2_	13.23	Egypt	0.906	8.987	10.416	59.0140, 261.2219, 279.2330	1
Oleic acid	Fatty acids	281.2486	C_18_H_34_O_2_	13.77	Greece	2.618	10.129	8.183	281.2489, 282.2519, 283.2552	1
13-Keto-octadeca-9*Z*,11*E*-dienoic acid (metabolites of α-linolenic acid)	Fatty acids	293.2122	C_18_H_30_O_3_	10.99 (10.29)	Greece	4.979	7.08	2.232	57.0347,113.0965, 179.1068, 195.1382, 293.2111	2a
9*R*-hydroxy-10*E*,12*Z*-octadecadienoic acid	Fatty acids	295.2279	C_18_H_32_O_3_	11.10 (12.00)	Greece	4.867	5.765	0.597	171.1020, 195.1382, 277.2161, 293.2116, 295.2266	2a
trans-EKODE-(E)-Ib	Fatty acids	309.2071	C_18_H_30_O_4_	9.35 (10.07)	Greece	2.791	4.512	1.979	137.0963, 139.1121, 155.1069, 167.1070, 171.1017	2a
(±)9-HpODE	Fatty acids	311.2228	C_18_H_32_O_4_	9.84 (11.83)	Greece	5.856	8.096	2.281	127.1112, 139.1121, 171.1018, 185.1176, 293.2117	2a
Oleoside methyl ester	Secoiridoids	403.1246	C_17_H_24_O_11_	3.08 (4.54)	Egypt	8.062	1.273	10.848	59.0140, 71.0140, 101.0242, 165.0554, 223.0607	3
8-epikingiside	Secoiridoids	403.1246	C_17_H_24_O_11_	4.03 (4.87)	Egypt	1.485	0.435	2.234	No fragmentation was observed in DDA mode due to very low abundance	3
Secoxyloganin	Secoiridoids	403.1246	C_17_H_24_O_11_	3.56 (4.61)	Egypt	7.514	1.025	9.474	59.0140, 71.0135, 101.0251, 121.0289, 165.0554	3
*p*-coumaroyl-6-secologanoside (Comselogoside)	Secoiridoids	535.1457	C_25_H_28_O_13_	4.61 (4.48)	Greece	4.045	5.239	1.198	69.0343, 145.0292, 163.0393, 205.0497, 265.0715	2a
Oleuropein	Secoiridoids	539.1770	C_25_H_32_O_13_	6.02	Egypt	2.354	0.25	3.049	59.0139, 101.0242, 181.0869, 275.0917, 307.0855	1
Dihydrooleuropein	Secoiridoids	543.2083	C_25_H_36_O_13_	5.41 (9.25 ^a^)	Egypt	2.957	0.072	3.72	59.0138, 71.0139, 151.0758, 313.1307, 377.1445	3
Verbascoside	Phenylpropanoids	623.1981	C_29_H_36_O_15_	4.97 (8.48 ^a^)	Egypt	9.278	0.552	11.386	113.0247, 161.0245, 315.1090, 461.1670, 623.1983	2a
Isoacteoside	Phenylpropanoids	623.1981	C_29_H_36_O_15_	5.48 (8.28 ^a^)	Egypt	8.979	0.542	10.984	135.0452, 161.0245, 315.1102, 461.1672, 623.1984	2b
β-Hydroxyverbascoside [Campneoside II]	Phenylpropanoids	639.1931	C_29_H_36_O_16_	4.41 (7.79 ^a^)	Egypt	8.015	1.738	11.251	151.0397, 161.0244, 179.0347, 459.1504, 621.1809	3
Unknown or in-source fragment of Campneoside II	Phenols	179.0350	C_9_H_8_O_4_	4.16	Egypt	2.637	0.151	3.372	123.0442, 137.0238, 151.0395, 179.0352	4

^a^ the compound was out of the application domain of the retention time prediction model.

## Supplementary Materials

The following are available online: Table S1: Target list of phenolic compounds, Table S2: Evaluation of scaling methods used by PLS-DA model, Figure S1: (a) EIC Chromatograms of the two mass features (±5 mDa); (b) Centroid mode spectra for the tR of 3.53 min; (c) MS/MS spectra of hydroxytyrosol (*m*/*z* 153.0558) eluting as same retention time as *m*/*z* 123.0452,
