# Supplementary material for: Authentication of Greek PDO Kalamata Table Olives: A Novel Non-Target High Resolution Mass Spectrometric Approach
Source: Molecules. 2020 Jun 24;25(12):2919. doi: 10.3390/molecules25122919 (PMC7355929; doi:10.3390/molecules25122919)
Supplement: Supplementary file 1 [file molecules-25-02919-s001.pdf]

molecules

MDPI

SUPPLEMENTARY MATERIAL

Authentication of *Greek PDO Kalamata* table olives: a novel non-target HRMS approach

Natasa P. Kalogiouri, Reza Aalizadeh, Marilena Dasenaki and Nikolaos S. Thomaidis*

Laboratory of Analytical Chemistry, Department of Chemistry, National and Kapodistrian University of Athens, Panepistimiopolis Zographou, 15771 Athens, Greece

* Correspondence:ntho@chem.uoa.gr; Tel: +30 210 7274317; Fax: +30 210 7274750

Table S1. Target list of phenolic compounds.

Compound Class	Compound	Molecular formula	[M-H]⁻ (m/z)	t_R (min)	Fragments (m/z)	Fragment elemental formula
flavonoids	Apigenin	$C_{15}H_{10}O_5$	269.0455	8.10	117.0340	C_8H_5O
					151.0031	$C_7H_3O_4$
	Epicatechin	$C_{15}H_{14}O_6$	289.0718	4.30	245.0819	$C_{14}H_{13}O_4$
					123.0452	$C_7H_7O_2$
					203.0714	$C_{12}H_{11}O_3$
	Luteolin	$C_{15}H_{10}O_6$	285.0405	7.30	133.0295	$C_8H_5O_2$
					151.0037	$C_7H_3O_4$
					175.0401	$C_{10}H_7O_3$
phenolic acids	Caffeic acid	$C_9H_8O_4$	179.035	1.40	135.0446	$C_8H_7O_2$
					134.0346	$C_8H_6O_2$
	Ferulic acid	$C_{10}H_{10}O_4$	193.0506	3.00	134.0373	$C_8H_6O_2$
					178.0272	$C_9H_6O_4$
	Gallic acid	$C_7H_6O_5$	169.0142	1.30	125.0244	$C_6H_5O_3$
					69.0344	C_4H_5O
					97.0295	$C_5H_5O_2$
	p-coumaric acid	$C_9H_8O_3$	163.0401	2.60	119.0502	C_8H_7O
					93.0344	C_6H_5O
	Syringic acid	$C_9H_{10}O_5$	197.0455	1.40	123.008	$C_6H_3O_3$
					166.9976	$C_7H_3O_5$
	Homovanillic acid	$C_9H_{10}O_4$	181.0491	1.48	122.0373	$C_7H_6O_2$
					137.0608	$C_8H_9O_2$
					154.0272	$C_7H_6O_4$
	Hydroxytyrosol	$C_8H_{10}O_3$	153.0557	3.50	123.0451	$C_7H_7O_2$
					95.0502	C_6H_7O
	Tyrosol	$C_8H_{10}O_2$	137.0608	4.10	119.0495	C_8H_7O
					107.0496	C_7H_7O
					93.034	C_6H_5O
	Vanillin	$C_8H_8O_3$	151.0401	4.70	136.0158	$C_7H_4O_3$
					108.0217	$C_6H_4O_2$
					92.0268	C_6H_4O
lignans	Pinoresinol	$C_{20}H_{22}O_6$	357.1344	6.49	151.0401	$C_8H_7O_3$
					136.0166	$C_7H_4O_3$
secoridoids	Oleuropein	$C_{25}H_{32}O_{13}$	539.1722	5.96	89.0244	$C_3H_5O_3$
					275.0925	$C_{15}H_{15}O_5$
					307.0823	$C_{15}H_{15}O_7$

Myricetin (IS)	$C_{15}H_{10}O_8$	317.0303	6.10	151.0031	$C_7H_3O_4$
				178.998	$C_8H_3O_5$

Table S2. Evaluation of scaling methods used by PLS-DA model.

Method	R^2X	R^2Y	Q^2	RMSEE	RMSEP	Classification Total Accuracy			Score
						Train set	Test set	Cross validation	
Mean Centering	0.867	0.971	0.966	0.0711	0.0921	1.00	1.00	1.00	0.931
Autoscaling	0.676	0.988	0.979	0.0528	0.0830	1.00	1.00	1.00	0.913
Range scaling	0.739	0.991	0.984	0.0466	0.0805	1.00	1.00	1.00	0.925
Pareto scaling	0.815	0.981	0.975	0.0674	0.0745	1.00	1.00	1.00	**0.932**
Vast	0.555	0.987	0.955	0.0563	0.0992	1.00	1.00	1.00	0.884
Level	0.736	0.989	0.976	0.0519	0.0776	1.00	1.00	1.00	0.923
Mean Centering Log2	0.972	0.978	0.971	0.0727	0.4880	1.00	0.215	0.831	0.810
VSN	0.931	0.034	0.0360	0.4701	0.5020	0.412	0.215	0.369	0.397
PQN	0.922	0.942	0.914	0.0745	0.0859	1.00	1.00	1.00	0.925

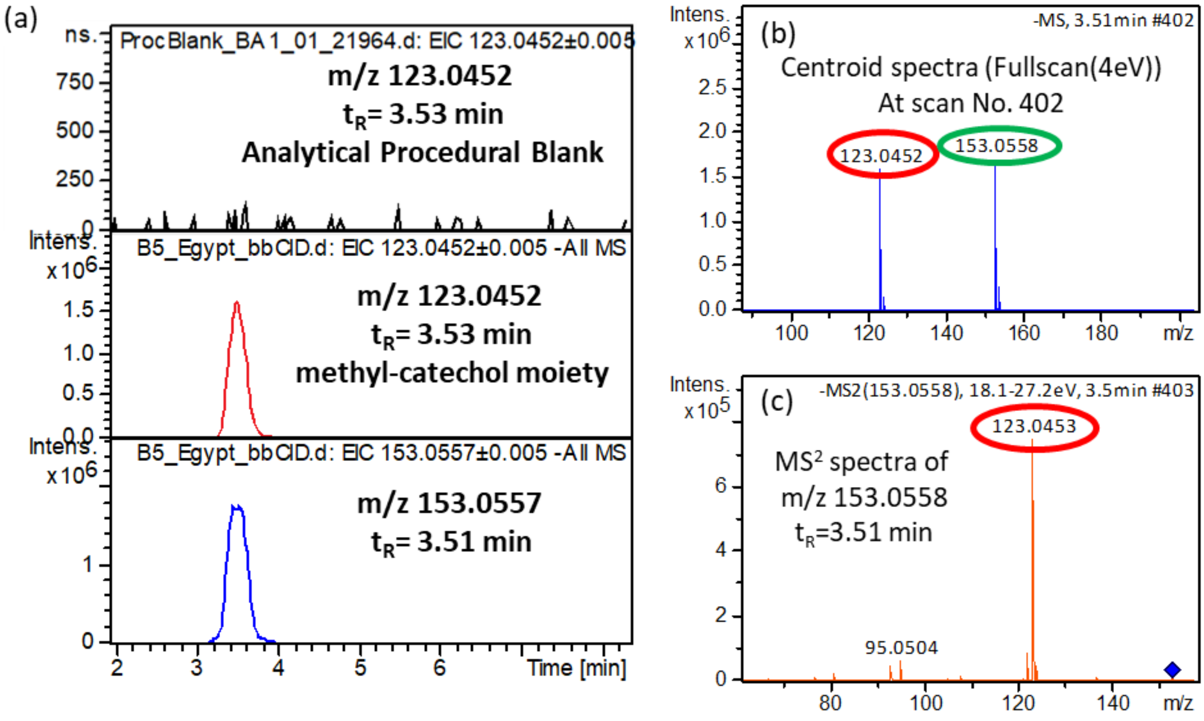

Figure S1. (a) EIC Chromatograms of the two mass features (±5 mDa); (b) Centroid mode spectra for the t_R of 3.53 min; (c) MS/MS spectra of hydroxytyrosol (m/z 153.0558) eluting as same retention time as m/z 123.0452.
